# Financial toxicity in hematological malignancies: a systematic review

**DOI:** 10.1038/s41408-022-00671-z

**Published:** 2022-04-22

**Authors:** Evguenia Ouchveridze, Rahul Banerjee, Aakash Desai, Muhammad Aziz, Wade Lee-Smith, Hira Mian, Katherine Berger, Brian McClune, Douglas Sborov, Muzaffar Qazilbash, Shaji Kumar, Ghulam Rehman Mohyuddin

**Affiliations:** 1 Department of Hematological Malignancies and Cellular Therapeutics, Kansas University Medical Center, Kansas, KS USA; 2 Division of Hematology/Oncology, Department of Medicine, University of California San Francisco, San Francisco, CA USA; 3 Division of Hematology, Mayo Clinic, Rochester, MN USA; 4 Mulford Health Science Library, University of Toledo, Toledo, OH USA; 5 Department of Oncology, McMaster University, Hamilton, ON Canada; 6 Patient Advocate, University of Hartford, West Hartford, CT USA; 7 Division of Hematology and Hematological Malignancies, Huntsman Cancer Institute, University of Utah, Salt Lake City, UT USA; 8 Division of Transplant, University of Texas MD Anderson Cancer Center, Houston, TX USA

**Keywords:** Quality of life, Haematological cancer

## Abstract

Hematologic malignancy outcomes have remarkably improved in the past decade with further advancement expected in future years. However, the detrimental effects of financial toxicity (FT) on patients with hematologic malignancies, because of both diagnoses and subsequent treatments, have not been studied comprehensively. We performed a systematic review of all studies reporting FT as a primary or secondary outcome among adult or pediatric patients with hematological malignancies. A total of 55 studies met the inclusion criteria for analysis. Across studies, 20–50% of patients reported some form of FT, including loss of work productivity, food and transportation costs, and depletion of savings. Younger age, lower-income level, unemployment, and rural residence were the most commonly identified risk factors for FT. Two studies looked at survival outcomes, with one reporting improvement in survival with a decrease in financial toxicity. However, significant heterogeneity in FT definitions was found between countries and payor systems. Only half of the studies (51%, *n* = 28) used validated survey instruments such as the COST assessment. The present systematic review identified that FT is common in patients with hematological malignancies and may be associated with poorer outcomes. However, studies of FT generally use non-standardized methods with cross-sectional analyses rather than longitudinal, prospective assessments. Further work is needed to standardize FT reporting and investigate measures to alleviate FT among patients with hematologic malignancies.

## Introduction

Financial toxicity (FT) is a source of strain for patients and caregivers during cancer therapy [1–5]. While exact definitions may vary, FT generally refers to the detrimental effects of cancer-directed therapy caused by out-of-pocket costs and lost productivity. FT is a patient-reported outcome that can be affected by many variables including insurance or payor coverage, geographic location, or cultural and personal factors [6, 7].

FT is particularly pronounced among patients with hematologic malignancies [8, 9]. The reasons for this are manifold. Patients with hematologic malignancies often face unique symptoms and quality of life (QOL) challenges which in turn interfere with their livelihood [10]. Additionally, certain hematologic malignancies such as acute leukemia are disproportionately managed in the inpatient setting with higher out-of-pocket costs. Finally, in diseases that are predominantly managed in the outpatient setting such as chronic myelogenous leukemia (CML), drug prices for oral small-molecule inhibitors remain unsustainably high [11, 12]. Understanding the prevalence of FT and its impact on outcomes represents an important step in devising future strategies for optimizing FT, as FT has been associated with poorer survival in other cancers [13].

There remains, however, a paucity of studies within malignant hematology that seek to identify risk factors, effects, and/or outcomes for FT. To better characterize FT in this population, we conducted a systematic review of studies investigating this topic in malignant hematology.

## Methods

There was no external funding for this review.

### Search strategy

One author (WL-S) developed a comprehensive search strategy of database-specific subject vocabulary (where available) and truncated keyword and phrase searches for the concept of financial toxicity and hematological malignancies. On 10 May, 2021, the search was executed in MEDLINE (PubMed, National Center for Biotechnology Information), Embase (Embase.com, Elsevier), Cochrane Central Register of Controlled Trials (Cochrane Library, Wiley), Web of Science Core Collection (Clarivate), and the following databases on the EBSCOhost platform: EconLit, Business Source Complete, Health Source: Nursing/Academic Edition, Psychology & Behavioral Sciences Collection, AP PsycINFO, and CINAHL Plus with Full Text. Example search strategies using these databases are depicted in Supplementary Tables 1–3. Records were exported to EndNote 20 (Clarivate, Philadelphia, Pennsylvania, USA) and deduplicated by software algorithm and manual inspection. Two independent reviewers (EO, RB) screened all studies, and any conflicts in eligibility (as defined below) were resolved through mutual discussion. This systematic review was performed according to the Preferred Reporting Items for Systematic Reviews and Meta-Analyses (PRISMA) recommendations [14].

Our search strategy included all studies (whether retrospective or prospective) where FT was directly investigated using qualitative or quantitative methods including surveys or interviews of patients and/or caregivers. Studies analyzing only institutional or systemic costs were excluded, as were reviews and editorials. Both adult and pediatric studies were included, and our search strategy was not restricted by language. All studies published on or before May 10, 2021 were considered.

For studies that met preliminary criteria for inclusion, we evaluated the focus on FT as well as on hematologic malignancies. Specifically, studies that only assessed FT as a component of a broader QOL instrument (without any further FT-specific delineation or discussion) were excluded. Similarly, studies were excluded if patients with blood cancers (leukemia, lymphoma, myeloma, or myeloproliferative neoplasm) constituted fewer than 25% of surveyed patients or if such cancer-specific data were not provided.

### Data collection

Three authors (EO, RB, and AD) performed and verified all data extraction for studies that were included in our systematic review. Extracted data were tabulated using Microsoft Excel (Microsoft, Redmond, Washington, United States). Baseline information for each study included the following: percent of subjects enrolled with hematologic malignancies, specific subtypes of hematologic malignancies included, number of patients enrolled, age distribution (pediatric or adult), geographic region or country represented, whether enrolled patients had undergone stem cell transplantation (SCT) and whether patients were in the active treatment versus survivorship setting.

Information on how FT was defined in each study was collected. Specific parameters included whether patient and/or caregiver FT was assessed as well as whether FT incorporated direct and/or indirect costs. As previously defined in the literature, direct costs included out-of-pocket (OOP) costs for cancer treatment including medication, office visits, hospitalization, and/or diagnostic studies [15]. Indirect costs included loss of work productivity (as measured by work hours or income), depletion of savings accounts, use of retirement funds, borrowing of money or use of credit cards, liquidation of assets or selling of property, and any other steps taken to cover the costs of cancer treatment. Living-related expenses while receiving cancer care (e.g., transportation, food, and housing) were also included as indirect costs [16].

Other extracted data included any information related to the repercussions of FT, including QOL impairments or worsened mental health. Finally, if studies offered an analysis of risk stratification for incurring FT, relevant data was collected. Where appropriate, subgroups were compared using chi-squared testing with a *p*-value of <0.05 determining statistical significance using SPSS (IBM Corporation, Armonk, New York, United States of America).

Further details on FT from individual studies were described in our manuscript by stratifying for the income level of countries. Income levels for respective countries were defined according to the World Bank Country and Lending Groups classification [17]. For the 2022 fiscal year, low-income countries were defined as those with a gross national income (GNI) per capita of $1045 or less in 2020; lower-middle-income countries were those with a GNI per capita between $1046 and $4095; upper-middle-income economies were those with a GNI per capita between $4096 and $12,695; and high-income economies were those with a GNI per capita of $12,696 or more.

Several studies from high-income countries and low-middle-income countries were arbitrarily selected to highlight specific results pertaining to FT.

## Results

**Fig. 1:**
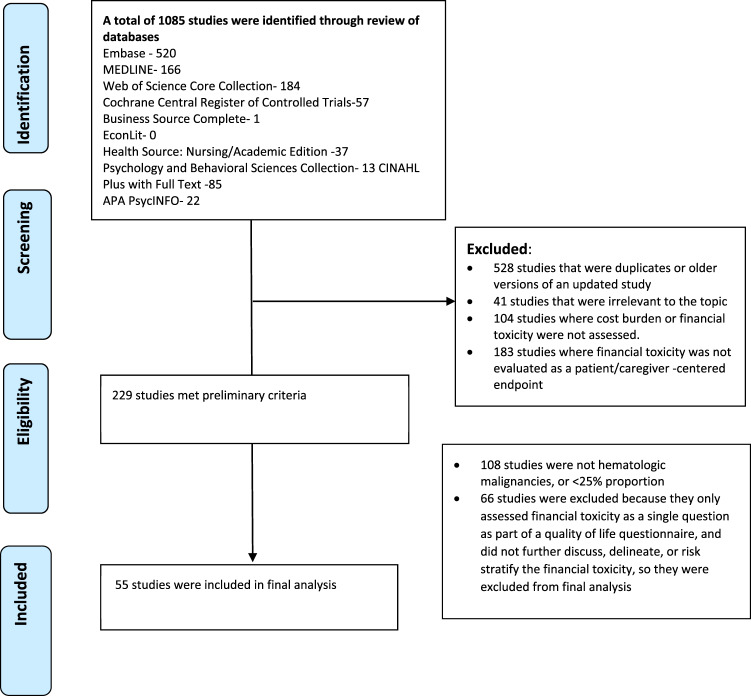
Flow diagram of study selection. The process of short-listing final list of included studies is highlighted.

As shown in Fig. 1, 229 out of 1085 screened studies assessed FT among patients with cancer. Of the 229 studies that met preliminary criteria for inclusion, 66 (29%) included FT only as a component of a larger QOL assessment without further description. In another 108 (47%) screened studies, the proportion of patients with hematologic malignancies was either not quantified or was less than 25%. A total of 55 studies satisfied all inclusion criteria and were thus further analyzed.

### Study characteristics

Table 1 highlights patient characteristics within the 55 included studies. Forty-two studies (76%) exclusively analyzed patients with hematologic malignancies. Studies of pediatric populations comprised 29% (*n* = 16) of studies, while studies focusing on SCT recipients comprised 22% (*n* = 12) of studies. The majority of studies (52%, *n* = 29) were based in North America; in contrast, only a single included study analyzed patients from Africa.

**Table 1:** Characteristics of patients in included studies.

	*n (%)*
Percent of subjects with hematologic malignancy	
100% of subjects with hematologic malignancies	42 (76.4%)
>25%, <100% subjects with hematologic malignancies	13 (23.6%)^a^
Specific cancers represented	
Leukemia only	16 (29.1%)
Lymphoma only	4 (7.3%)
Multiple myeloma only	5 (9.1%)
Myeloproliferative neoplasm only	1 (1.8%)
Multi-disease	29 (52.7%)
BMT or cell therapy	12 (21.8%)
Survivorship, or >5 years out from treatment	8 (14.5%)
Pediatric subjects	16 (29.1%)
Median number of subjects per study	162
Financial toxicity to the caregiver	19 (34.5%)^b^
Geographic region	
North America	29 (52.7%)
Asia	16 (29.1%)
Oceania	5 (9.1%)
Europe	3 (5.5%)
Africa	1 (1.8%)

^a^In these 13 studies (23.6%) the remainder of subjects who did not have a hematologic malignancy represented either solid organ malignancies or a benign hematologic cause for bone marrow transplant.

^b^Of these 19 studies that assessed financial toxicity to the caregiver, two studies (10.9%) assessed financial toxicity to both the caregiver and the patient, and in three studies (15.7%) the caregiver was for an adult patient.

Table 2 highlights the FT-relevant methods employed by the 55 included studies. While 78% of studies (*n* = 43) used at least one FT-specific survey instrument, the remaining 12 studies used open-ended interviews to assess FT. Of the 43 studies that employed a survey instrument, 35% (*n* = 15) used custom survey-specific questionnaires without any reported validation. Of the studies that did employ validated survey instruments, the most commonly used tool was the Comprehensive Score for Financial Toxicity (COST) assessment [18] (*n* = 8, 14% of studies analyzing FT). Twenty-six studies (47%) specifically analyzed indirect costs as outlined in Table 3 [6, 19–43]. Nineteen studies (35%) specifically analyzed caregiver FT, primarily in the pediatric setting.

**Table 2:** Methods used in included studies.

		Studies (*n*)	Percent (%)
Type of instrument			
	Custom questionnaire	15	27.3%
	Single validated questionnaire	9	16.3%
	Multiple validated questionnaires	19	34.5%
	Interviews alone	12	21.8%

**Table 3:** Specific examples of financial toxicity used by studies.

	Abbasnezhad, M., et al.	Albelda, R., et al.	Bona, K., et al.	Carey, M., et al.	Flucehl, M. N., et al	Fortune, E. E., et al.	Goodwin, J. A., et al.	Gupta, S., et al.	Hall, A. E., et al.	Harrison, C., et al.	Huntington, S. F., et al.	Islam, M. Z., et al.	Khera, N., et al. (2017)	Klassen, A. F., et al.	Knight, T. G., et al.	Limburg, H., et al.	Maheshwari, S., et al.	McGrath, P.	Meehan, K.R., et al.	Mostert, S., et al.	Muffly, L. S., et al.	Pearse, W. B., et al.	Poudyal, B. S., et al.	Ren, Y and X. Li	Sneha, L. M., et al.	Warner, E. L., et al.
Productivity loss-income or hours decreased, job change or loss			x	x	x		x	x		x		x		x		x	x			x		x			x	x
Using/depleting retirement funds													x													
Using college funds			x																							
Allowing life insurance policy to lapse			x																							
Depleting savings	x			x		x			x		x				x										x	
Borrowing money				x		x					x		x										x		x	
Utilizing credit card/lines of credit							x																			
Liquidating assets, selling land						x																	x		x	
Not enough money at end of the month	x	x							x				x		x											
Food costs			x									x			x		x	x	x		x			x	x	
Housing costs			x															x			x			x	x	
Utilities/electricity			x	x									x						x		x					
Transportation, gas costs					x		x	x							x		x	x						x	x	
Relocation					x																					
Disability/unemployment						x	x																			

Refs. [6, 19–37,19–37,39–43,].

### Risk factors for FT

A total of 18 studies (33%) stratified risk factors of incurring FT. The most commonly assessed risk factor was age, in 10 out of 18 studies (56%); in particular, seven studies identified younger age as a risk factor for FT [29, 44–49]. Age was followed closely by pre-existing economic factors such as employment status, lower-income level, and a limited ability to provide basic needs. Eight studies (44%) identified these variables as risk factors for FT [29, 44, 46, 47, 49–51]. Similarly, several studies reported higher FT among rural patients than among patients from urban/suburban areas [23, 30, 41–43, 49]. Table 4 highlights risk factors for incurring FT in more detail.

**Table 4:** Risk factors for incurring financial toxicity.

	Age	Income, employment status, ability to provide basic needs	Rural	Sex	Time since diagnosis	Education	Race	Marital status
Bala-Hampton, J. E., et al. [44]	x	x						
Flucehl, M. N., et al. [23]			x					
Huang, I. C., et al. [50]	x	x				x		
Huntington, S. F., et al. [29]	x	x			x			x
Islam, M. Z., et al. [30]	x		x					
Jones, S. M. W., et al. [45]	x			x				
Jones, W. C., et al. [46]	x	x			x			x
Khera, N., et al. [31]		x						
Kim, S. H., et al. [69]								
Knight, T. G., et al. [47]	x	x					x	
Poudyal, B. S., et al. [6]								
Priscilla, D., et al. [51]	x	x		x				
Ren, Y. and X. Li. [41]			x					
Sidi Mohamed El Amine, B, et al [70]								
Sneha, L. M., et al. [42]			x					
Van Der Poel, M. W. M., et al. [48]	x							
Warner, E. L., et al. [43]			x		x			
Warsame, R. M., et al. [49]	x	x	x	x		x		x

### Association of FT with outcomes

Two studies assessed the association of FT with survival. In one study from China of patients with acute myelogenous leukemia (AML), the authors showed an association between increased health insurance coverage and improved survival due to decreased FT-related treatment abandonment [52]. In another study, however, no statistically significant difference in survival with regard to FT scores was found [47]. Fifteen studies reported an association between increased FT and other components of QOL [19, 20, 22, 25, 27, 33, 44, 47, 49, 50, 53–57]. Most commonly, increased FT was associated with increased psychological distress or depression.

### Interventions to mitigate FT

A total of five studies (two retrospective, three prospective) investigated an intervention to mitigate the FT of a hematologic malignancy. Table 5 highlights the characteristics of these studies.

**Table 5:** Interventions to alleviate financial toxicity.

Study	Number of subjects	Intervention	Outcomes
Knight TG., et al. Prospective, intervention study [71]	105	Group 1 met with a nurse navigator, a clinical pharmacist, and a financial planner to identify and address gaps in coverage, provide financial and budgeting assistance. Group 2 was standard assistance arm.	After adjusting for insurance, race, and age at survey, the risk of death with the intervention was 0.47 times the risk of death in those without the intervention (95% CI 0.23–0.98, *p* = 0.043).
De Souza JA., et al. Prospective, intervention study [72]	308	Co-pay assistance from the Patient Access Network Foundation, with assessment of financial toxicity over a period of 3 months.	89% had an improvement in financial toxicity over the 3 months.
Albelda R, et al. Retrospective analysis [20]	171	Analysis of the effect of paid leave in the BMT population, on financial burden at 6 months post-transplant.	Paid leave improved financial burden in the post-transplant period when looking at three separate measures of FT (*p* < 0.05).
Sidana, S., et al. Prospective analysis [73]	123	Assessing financial burden in patients enrolled on clinical trial (*n* = 34, 28%) versus not on clinical trial (*n* = 89, 72%) over the first year of treatment.	Patients on clinical trials (CT) reported less need for taking extended time off from work (22% CT vs 46% non-CT *p* = 0.02). Financial burden was found to be lower in the CT group but differences not statistically significant.
Hong, D., et al. Retrospective analysis [52]	474	Analysis of the rates of treatment abandonment in a province in China, before and after adoption of increased government insurance aid policies.	Abandonment of treatment decreased from 40% (6/15) to 0% (0/6) after new insurance policies were set in place.

### Representative FT studies from high-income countries

A study that surveyed 32 United States AML patients showed that 69.2% did not have enough money to cover the cost of treatments, 65.4% had greater than anticipated out-of-pocket expenses, and 62% stated that their financial stress came from their cancer treatment [44].

A survey was conducted on FT related to SCT in the United States in 45 families of children who underwent transplants in the US [21]. Of the families surveyed, 12% of parents reported quitting jobs or being laid off due to their child’s transplant and 20% of families lost >40% of their annual income, with the greatest proportion of income loss in low-income families. Additionally, 26% of families were unable to pay bills due to treatment costs, and 38% of families reported insecurity in food, energy, or housing during the post-transplant period, again, with a higher proportion in low-income families [21].

A study examined the financial needs of 2198 children and young adults with cancer (36% of patients with lymphoma and 9% with lymphoma) who applied for financial assistance from the Family Reach Foundation between 2010 and 2015 in the United States [39]. Identified causes of the need for financial assistance were largely non-medical, and included rent, mortgage, food, and auto payments. These were second to medical costs such as copays, health insurance premiums, travel to the hospital, and lodging. Rent and mortgage accounted for 62% of the burden [39].

A United Kingdom-based study looked at the repercussions on employment by surveying 286 patients with myeloproliferative neoplasms (MPNs) [28]. Patients reported a high impact of disease on their ability to work: 26% reduced their work hours, 13% voluntarily left their job, 12% took early retirement, 9% took a lower-paying job, and 9% went on disability [28].

A pediatric study of 354 patients in the United States looked at the repercussions of living in a rural versus urban environment for pediatric patients with a diagnosis of acute lymphoblastic leukemia (ALL) (44%) or AML (5%) [23]. Those who lived in rural or remote areas (>1 h travel) had a higher number of workdays missed, compared to urban counterparts, in the month following their child’s initial diagnosis. This difference did not persist beyond 6 months post-diagnosis. One-third of caregivers reported changing jobs or quitting as a result of their child’s cancer diagnosis, and rural parents had greater out-of-pocket travel-related expenses and significantly higher self-reported financial burdens than their urban counterparts.

A study of CML patients in the United States, which defined financial burden as depleting savings or utilizing money from retirement funds, showed there was a significant association between financial burden and suboptimal medication adherence (*p* < 0.01). The investigators showed that 14% of patients postponed filling prescriptions to reduce healthcare costs and 31% of patients were found to have suboptimal CML medication adherence. Additionally, it was shown that 16% of patients postponed doctor’s appointments due to help reduce healthcare costs, and 23% postponed complementary treatment such as psychological counseling [53].

A study out of the Netherlands compared survivors of diffuse large B cell lymphoma (DLBCL) with age-matched controls. An EORTC-QLQ C30 questionnaire was completed by 307 subjects; survivors of non-Hodgkin lymphoma (NHL) in the age range 18–59 experienced significantly more financial difficulties in comparison with an age-matched normative population. There was such a difference seen in older survivors as well, but this difference was not significant [48].

Another study in the United States of 2811 survivors of childhood malignancies, of whom 57.8% had hematologic malignancies, reported financial hardship in 22.4% of survivors, with the determinants of financial hardship identified as lower educational attainment, lower household income, and older age (>40 years) at evaluation. There was also a significant association between financial hardship and physical and psychological symptoms, including anxiety, depression, and suicidal ideation. Financial hardship was also statistically significantly associated with difficulty in acquiring health and life insurance, and poor retirement planning [50].

A study of 182 caregivers of adult patients in Australia showed that 40% of caregivers had to take time off work, 29% reported a drop in income, 19% used up their savings, 14% had difficulty with bills, and 4.8% had to borrow money [22].

A separate study of caregivers of 354 pediatric subjects with acute leukemia in the United States showed that 36% of caregivers reported quitting or changing jobs due to a child’s cancer diagnosis and that caregivers missed a mean of 17.3 days of work in the first month after diagnosis [23].

### Representative FT studies from lower-middle-income countries

A questionnaire was filled out by 70 families of children in India diagnosed with acute leukemia and on active treatment, to assess out-of-pocket expenses and the financial burden of their child’s diagnosis [42]. According to the survey results, 38% of parents had to take on additional jobs and overtime to avoid debt and financial crisis, 62% of households used up their savings, and 68% went into debt. The results indicated that 55% of households borrowed money, 25% sold properties and assets, and one family abandoned treatment due to FT. Of households with multiple children, 50% had to restrict spending on the needs of other children to afford care for the child with cancer. The study found that food, travel, and accommodation accounted for two-thirds of total expenses incurred during hospital admissions [42].

In another 70-family pediatric study out of India, non-medical expenses accounted for 46% of the monthly income of parents from rural areas and 22% of those from urban areas. Food and travel expenses were a major contributing factor to the financial burden, and households with multiple children had to restrict spending on the needs of other children due to the cost of treatment [58].

In a study of 390 pediatric patients in Indonesia, it was reported that 69% of families’ income decreased due to parental work hours lost, and in 18% of cases parents were forced to postpone or withdraw their children from treatment due to financial difficulties [38].

## Discussion

Our analysis represents the first comprehensive systematic review of FT among patients with hematological malignancies. As expected, given the intensive nature of blood cancers and their treatments, we found FT to be a prevalent and persistent problem. Specifically, ~20–50% of all patients reported some form of FT, regardless of disease or country. We identified several risk factors for incurring FT, including extremes of age, pre-existing negative economic factors, and living in a rural rather than urban area. Several studies identified FT as a risk factor for impaired psychosocial well-being and even decreased survival. Of note, our systematic review identified decreased medication adherence (or outright abandonment of treatment) as risk of FT; however, interventions to address FT or its downstream effects on medication adherence were scarce in nature and predominantly retrospective.

With regard to study methodology, only a minority of analyzed studies investigated FT in a dedicated manner rather than as a single component of a broader QOL instrument. Several, but not all, studies incorporated indirect costs or caregiver FT as a component of their analyses. There was considerable heterogeneity in the definitions of FT between studies. This is unsurprising given that FT is a subjective measure affected by economic factors in the country of interest and socioeconomic factors in the patients of interest. Our findings are parallel to what has been observed regarding FT in solid tumors [59]. However, given that validated instruments such as the COST assessment now exist to capture these components [60], we suggest that consistent use of these tools and incorporation of a validated framework for quantitating FT may be helpful for future studies. The use of long-term, longitudinal measurements of FT, rather than snapshots of a single timepoint, may also better characterize the impact of cancer on patients’ (and their caregivers’) lives across stages. This is especially relevant given that patients with hematologic malignancies are living longer. Additionally, in survivors of hematologic malignancies, there is a persistence of financial distress despite no longer being on active treatment. Further studies are needed on how FT may impact QOL and long-term clinical outcomes.

Of note, we included all studies on financial toxicity from conception until 2021. Much has changed about the treatment of blood cancers during the last several decades, most prominently improved survival rates in several hematologic malignancies, as evidenced by analysis from large nationwide databases in the United States and abroad [61–65]. As summarized in Supplemental Table 1 which highlights various examples across several hematological malignancies, novel therapeutic options may have contributed to this trend. However, although these drugs may be more efficacious and tolerable, they are generally patent-protected and thus more expensive for patients and payers.

Another change pertinent to our review is the advent of new technologies or policies to potentially alleviate FT, for example, Medicaid expansion through the Affordable Care Act or online crowdfunding platforms such as GoFundMe [66–68]. Regardless of these steps, however, our review demonstrates the durability of FT as a patient-facing barrier in need of better recognition and management.

The strengths of our analysis include its emphasis on FT as described by both quantitative and qualitative methods and our inclusion of studies from both high-income and low-middle-income countries. However, our analysis has key limitations as well. Given our emphasis on the patient-centered endpoint of FT (as well as stark differences in annual incomes between nations), we excluded studies that analyzed objective measures of out-of-pocket costs using institutional or insurance databases. Similarly, to focus on the unique needs of patients with hematologic malignancies, we excluded studies where the proportion of patients with eligible cancers was low or not listed. These exclusions may have led to a bias toward studies of patients being treated at high-volume or academic centers. Furthermore, we did not search international databases such as SciELO, and hence our analysis may not have comprehensively included all FT studies. We qualitatively described the results of certain FT studies, but due to the heterogeneity of the data presented, a quantitative aggregate of the data was unable to be performed. The selection of which studies to include for further discussion was also done in an arbitrary manner.

In summary, we highlight the current state of the literature on FT in hematological malignancies and highlight potential areas for improvement. FT is a heterogeneous and subjective measure that is pervasive amongst patients with hematologic malignancies worldwide and affects physical, emotional, social, and financial well-being with long-lasting effects. Future methodological steps include the use of dedicated FT survey instruments such as the COST assessment and the incorporation of longitudinal assessments. More importantly, future steps for our field include prospective interventions to address the financial and logistical gaps that predispose vulnerable patients to worse outcomes.

## Supplementary information

Supplement

Checklist

## Data Availability

Template data collection forms, data extracted from included studies, and data used for all analyses are all available upon request to the corresponding author.
