# Supplementary material for: Financial toxicity in hematological malignancies: a systematic review
Source: Blood Cancer J. 2022 Apr 22;12(4):74. doi: 10.1038/s41408-022-00671-z (PMC9033803; doi:10.1038/s41408-022-00671-z)
Supplement: Supplementary file 1 — Supplement [file 41408_2022_671_MOESM1_ESM.pdf]

SUPPLEMENTARY TABLE 1: Examples of novel therapeutic options compared to older options, OS= overall survival, PFS= progression free survival.

Disease	Patent-protected drug	Older drug	Theoretical benefits
Multiple myeloma	Carfilzomib (2012)	Bortezomib (2003)	Less neuropathy in ENDEAVOR trial[1]
CML	Dasatinib (2006)	Imatinib (2001)	Fewer musculoskeletal AEs in DASISION trial[2]
Non-Hodgkin's lymphoma	Polatuzumab (2019)	Vincristine (1963)	Improved PFS in POLARIX trial[3]
Hodgkin's lymphoma	Brentuximab (2018)	Bleomycin (1973)	Improved PFS in ECHELON-1 trial[4]
AML	CPX-351 (2017)	Daunorubicin (1979) and cytarabine (1969)	Improved OS in Phase 3 trial[5]
CLL	Ibrutinib (2013)	Chlorambucil (1957)	Improved OS in RESONATE-2 trial [6]

References:

1. Dimopoulos MA, Goldschmidt H, Niesvizky R, et al. Carfilzomib or bortezomib in relapsed or refractory multiple myeloma (ENDEAVOR): an interim overall survival analysis of an open-label, randomised, phase 3 trial. *Lancet Oncol* 2017; **18**(10): 1327-37.
2. Cortes JE, Saglio G, Kantarjian HM, et al. Final 5-Year Study Results of DASISION: The Dasatinib Versus Imatinib Study in Treatment-Naive Chronic Myeloid Leukemia Patients Trial. *J Clin Oncol* 2016; **34**(20): 2333-40.
3. Tilly H, Morschhauser F, Sehn LH, et al. Polatuzumab Vedotin in Previously Untreated Diffuse Large B-Cell Lymphoma. *N Engl J Med* 2022; **386**(4): 351-63.
4. Straus DJ, Dlugosz-Danecka M, Connors JM, et al. Brentuximab vedotin with chemotherapy for stage III or IV classical Hodgkin lymphoma (ECHELON-1): 5-year update of an international, open-label, randomised, phase 3 trial. *Lancet Haematol* 2021; **8**(6): e410-e21.
5. Lancet JE, Uy GL, Newell LF, et al. CPX-351 versus 7+3 cytarabine and daunorubicin chemotherapy in older adults with newly diagnosed high-risk or secondary acute myeloid leukaemia: 5-year results of a randomised, open-label, multicentre, phase 3 trial. *Lancet Haematol* 2021; **8**(7): e481-e91.
6. Burger JA, Tedeschi A, Barr PM, et al. Ibrutinib as Initial Therapy for Patients with Chronic Lymphocytic Leukemia. *N Engl J Med* 2015; **373**(25): 2425-37.

Embase Search Strategy with comments (May 10, 2021, Embase.com platform, Elsevier)

No.	Summary	Results	Query
1	Financial Toxicity (FT)	21734	'financial stress'/exp OR 'financial toxic*' OR 'financial distress*' OR 'financial burden*' OR 'financial stress*' OR 'financial hardship*' OR 'financial strain*' OR 'financial difficult*' OR 'financial consequence*' OR (('out of pocket*' OR 'patient expen*' OR 'personal financ*' OR 'financial management*' OR 'financial management'/de OR 'financial impact*') AND ('patient report*' OR 'patient-reported outcome'/de OR 'patient burden*' OR 'caregiver burden*' OR 'caregiver burden'/exp OR 'quality of life' OR 'quality of life'/exp OR 'cost of illness' OR 'cost of illness'/exp OR 'mental stress'/exp OR 'mental stress*' OR 'psychological stress*' OR 'psychosocial stress*' OR 'psycho social stress*'))
2	Multiple Myeloma	94059	'multiple myeloma'/exp OR 'multiple myeloma*' OR 'kahler disease*' OR 'morbus kahler*' OR 'myeloma multiplex*' OR myelomatosis* OR 'plasma cell myeloma'
3	Lymphomas	412908	'lymphoma'/exp OR lymphoma* OR adenolymphoma* OR 'lymph node tumor*' OR 'lymph node tumour*' OR 'lymphocytic tumor*' OR 'lymphocytic tumour*' OR 'lymphoid malignanc*' OR 'malignant lymphoid neoplasm*'
4	Leukemias	525432	'leukemia'/exp OR leukemia* OR aleukemia* OR aleukaemia* OR hemoblastoma* OR leukaemia* OR leucemia*
5	Any hematological cancer	766657	'hematologic malignancy'/exp OR 'hematologic malignanc*' OR 'haematologic malignanc*' OR 'hematological malignanc*' OR 'haematological malignanc*' OR 'hematological cancer*' OR 'haematological cancer*'

6	FT AND MM	141	#1 AND #2
7	FT AND Lymphoma	254	#1 AND #3
8	FT AND leukemia	367	#1 AND #4
9	FT AND Any	619	#1 AND #5
10	Any of the above	926569	#2 OR #3 OR #4 OR #5
11	FT and Any of the above	654	#1 AND #10
12	exclude unwanted publication types	567	#11 NOT ('conference review'/it OR 'editorial'/it OR 'review'/it OR 'short survey'/it)
13	exclude unwanted study types	520	#12 NOT ('animal cell'/de OR 'animal experiment'/de OR 'animal model'/de OR 'case report'/de OR 'mouse model'/de OR 'practice guideline'/de OR 'systematic review'/de)

MEDLINE/PubMed Search Strategy (May 10, 2021, PubMed.gov platform, NCBI)

Search number	Query	Results	Time
1	Financial-toxic* OR financial-distress* OR financial-burden* OR "Financial Stress"[Mesh] OR financial-stress* OR financial-hardship* OR financial-strain* OR financial-difficult* OR financial-consequence* OR ((out-of-pocket* OR patient-expen* OR "Financing, Personal"[Mesh] OR Personal-financ* OR financial-management* OR financial-impact*) AND (patient-report* OR "Patient Reported Outcome Measures"[Mesh] OR patient-burden* OR "Caregiver Burden"[Mesh] OR caregiver-burden* OR "Quality of Life"[Mesh] OR quality-of-life* OR "Cost of Illness"[Mesh] OR cost-of-illness* OR mental-stress* OR "Stress, Psychological"[Mesh] OR psychological-stress* OR psychosocial-stress* OR psycho-social-stress*))	12,840	14:39:51
2	"Multiple Myeloma"[Mesh] OR Multiple-myeloma* OR Kahler-disease* OR morbus-kahler* OR myeloma-multiplex* OR myelomatosis* OR plasma-cell-myeloma*	55,033	14:39:57
3	"Lymphoma"[Mesh] OR Lymphoma* OR adenolymphoma* OR lymph-node-tumor* OR lymph-node-tumour* OR lymphocytic-tumor* OR lymphocytic-tumour* OR lymphoid-malignanc* OR malignant-lymphoid-neoplasm*	262,719	14:40:06
4	"Leukemia"[Mesh] OR Leukemia* OR aleukemia* OR aleukaemia* OR hemoblastoma* OR Leukaemia* OR leucemia*	350,565	14:40:11
5	"Hematologic Neoplasms"[Mesh] OR Hematologic-malignanc* OR haematologic-malignanc* OR hematological-malignanc* OR haematological-malignanc* OR hematological-cancer* OR haematological-cancer*	45,386	14:40:17
6	#2 OR #3 OR #4 OR #5	614,251	14:40:28
7	#1 AND #6	211	14:40:34

8	#7 NOT ("animals"[mesh] NOT "humans"[mesh])	210	14:40:52
9	#8 NOT ("case reports"[Publication Type] OR "comment"[Publication Type] OR "editorial"[Publication Type] OR "guideline"[Publication Type] OR "introductory journal article"[Publication Type] OR "meta analysis"[Publication Type] OR "news"[Publication Type] OR "retracted publication"[Publication Type] OR "review"[Publication Type] OR "systematic review"[Publication Type])	166	14:41:10

Web of Science Core Collection (searched 5 May 2021, Web of Science Platform, Clarivate)

Search	Results
You searched for:<br>**TOPIC:** (Financial-toxic* OR financial-distress* OR financial-burden* OR financial-stress* OR financial-hardship* OR financial-strain* OR financial-difficult* OR financial-consequence* OR ((out-of-pocket* OR patient-expen* OR Personal-financ* OR financial-management* OR financial-impact*) AND (patient-report* OR patient-burden* OR caregiver-burden* OR quality-of-life* OR 'cost of illness' OR mental-stress* OR psychological-stress* OR psychosocial-stress* OR psycho-social-stress*)))<br>*AND*<br>**TOPIC:** (Multiple-myeloma* OR Kahler-disease* OR morbus-kahler* OR myeloma-multiplex* OR myelomatosis* OR plasma-cell-myeloma* OR Lymphoma* OR adenolymphoma* OR lymph-node-tumor* OR lymph-node-tumour* OR lymphocytic-tumor* OR lymphocytic-tumour* OR lymphoid-malignanc* OR malignant-lymphoid neoplasm* OR Leukemia* OR aleukemia* OR aleukaemia* OR hemoblastoma* OR Leukaemia* OR leucemia* OR He matologic-malignanc* OR haematologic-malignanc* OR hematological-malignanc* OR haematological-malignanc* OR hematological-cancer* OR haematological-cancer*)	184
**Refined by:** [excluding] **DOCUMENT TYPES:** (REVIEW OR EDITORIAL MATERIAL OR NEWS ITEM)	
Timespan: All years. Indexes: SCI-EXPANDED, SSCI, A&HCI, CPCI-S, CPCI-SSH, ESCI. (from Web of Science Core Collection)	

Cochrane Central Register of Controlled Trials (searched 5 May 2021, Cochranelibrary.com, Wiley)

	Search	
ID	Financial-toxic* OR financial-distress* OR financial-burden* OR financial-stress* OR financial-hardship* OR financial-strain* OR financial-difficult* OR financial-consequence*	Hits
#1	out-of-pocket* OR patient-expen* OR Personal-financ* OR financial-management* OR financial-impact*	1031
#2	patient-report* OR patient-burden* OR caregiver-burden* OR quality-of-life* OR 'cost of illness' OR mental-stress* OR psychological-stress* OR psychosocial-stress* OR psycho-social-stress*	1411

#3	#2 AND #3	135995
#4	#1 OR #4	432
#5	Multiple-myeloma* OR Kahler-disease* OR morbus-kahler* OR myeloma-multiplex* OR myelomatosis* OR plasma-cell-myeloma*	1423
#6	Lymphoma* OR adenolymphoma* OR lymph-node-tumor* OR lymph-node-tumour* OR lymphocytic-tumor* OR lymphocytic-tumour* OR lymphoid-malignanc* OR malignant-lymphoid-neoplasm*	5180
#7	Leukemia* OR aleukemia* OR aleukaemia* OR hemoblastoma* OR Leukaemia* OR leucemia*	12731
#8	Hematologic-malignanc* OR haematologic-malignanc* OR hematological-malignanc* OR haematological-malignanc* OR hematological-cancer* OR haematological-cancer*	15281
#9	#6 OR #7 OR #8 OR #9	2471
#10	#5 AND #10	29554
#11		57

EBSCOhost Databases (searched 10 May 2021, EBSCOhost platform, EBSCO)

((Financial-toxic* OR financial-distress* OR financial-burden* OR financial-stress* OR financial-hardship* OR financial-strain* OR financial-difficult* OR financial-consequence* OR ((out-of-pocket* OR patient-expen* OR Personal-financ* OR financial-management* OR financial-impact*) AND (patient-report* OR patient-burden* OR caregiver-burden* OR quality-of-life* OR 'cost of illness' OR mental-stress* OR psychological-stress* OR psychosocial-stress* OR psycho-social-stress*)))) AND ((Multiple-myeloma* OR Kahler-disease* OR morbus-kahler* OR myeloma-multiplex* OR myelomatosis* OR plasma-cell-myeloma* OR Lymphoma* OR adenolymphoma* OR lymph-node-tumor* OR lymph-node-tumour* OR lymphocytic-tumor* OR lymphocytic-tumour* OR lymphoid-malignanc* OR malignant-lymphoid-neoplasm* OR Leukemia* OR aleukemia* OR aleukaemia* OR hemoblastoma* OR Leukaemia* OR leucemia* OR Hematologic-malignanc* OR haematologic-malignanc* OR hematological-malignanc* OR haematological-malignanc* OR hematological-cancer* OR haematological-cancer*))	
Limiters - Scholarly (Peer Reviewed) Journals	
**Business Source Complete**	1
**EconLit**	0
**Health Source: Nursing/Academic Edition**	37

**Psychology and Behavioral Sciences Collection**	13
**CINAHL Plus with Full Text**	85
**APA PsycINFO**	22

LIST OF ALL INCLUDED STUDIES:

1. Abbasnezhad, M., et al. (2015). "Cancer Care Burden among Primary Family Caregivers of Iranian Hematologic Cancer Patients." Asian Pac J Cancer Prev 16(13): 5499-5505.
2. Al Qadire, M., et al. (2020). "Burden among Parents of Children with Cancer in Jordan: Prevalence and Predictors." Cancer Nursing 43(5): 396-401.
3. Albelda, R., et al. (2019). "Relationship between paid leave, financial burden, and patient-reported outcomes among employed patients who have undergone bone marrow transplantation." Quality of Life Research 28(7): 1835-1847.
4. Bala-Hampton, J. E., et al. (2017). "Perceived Economic Hardship and Distress in Acute Myelogenous Leukemia." Journal of Oncology Navigation & Survivorship 8(6): 258-264.
5. Bona, K., et al. (2015). "Prevalence and impact of financial hardship among New England pediatric stem cell transplantation families." Biol Blood Marrow Transplant 21(2): 312-318.
6. Buzaglo, J. S., et al. (2017). "Medication Adherence Among Patients with Chronic Myeloid Leukemia: The Impact of Financial Burden and Psychosocial Distress." Journal of Oncology Navigation & Survivorship 8(4): 168-175.
7. Carey, M., et al. (2012). "Financial and social impact of supporting a haematological cancer survivor." European Journal of Cancer Care 21(2): 169-176.
8. Clark, S. E., et al. (2020). "Predictors of tyrosine kinase inhibitor adherence trajectories in patients with newly diagnosed chronic myeloid leukemia." Journal of Oncology Pharmacy Practice: 1078155220970616.
9. De Souza, J. A., et al. (2017). "Evaluating financial toxicity (FT) interventions." Journal of Clinical Oncology 35(15).
10. Dhir, V., et al. (2019). "Quality of life and caregiver burden inmultiple myeloma and lymphoma patients and their caregivers undergoing outpatient autologous stem cell transplantation compared to inpatient transplantation." Supportive Care in Cancer 27(1): S223-S224.
11. Flucehl, M. N., et al. (2012). "The impact of geography on the burden of care for pediatric oncology patients and their families." Pediatric Blood and Cancer 59(6): 1024.
12. Fortune, E. E., et al. (2020). "Financial burden is associated with postponing care anddecreasing physical and emotional quality of life among patientswith multiple myeloma and chronic lymphocytic leukemia." Blood 136(SUPPL 1): 45.
13. Goodwin, J. A., et al. (2013). "Personal financial effects of multiple myeloma and its treatment." Cancer Nurs 36(4): 301-308.

14. Gupta, S., et al. (2018). "Assessing the Effect of Adherence on Patient-reported Outcomes and Out of Pocket Costs Among Patients With Multiple Myeloma." Clinical Lymphoma, Myeloma and Leukemia 18(3): 210-218.
15. Hall, A. E., et al. (2015). "What do haematological cancer survivors want help with? A cross-sectional investigation of unmet supportive care needs." BMC research notes 8: 221.
16. Hall, A. E., et al. (2016). "Prevalence and associates of psychological distress in haematological cancer survivors." Supportive Care in Cancer 24(10): 4413-4422.
17. Harrison, C., et al. (2017). "Patient perceptions on the symptomatic and economic burden of myeloproliferative neoplasms." British Journal of Haematology 176: 23-24.
18. Hong, D., et al. (2016). "A 10-year follow-up survey of treatment abandonment of children with acute myeloid leukemia in Suzhou, China." Journal of Pediatric Hematology/Oncology 38(6): 437-442.
19. Huang, I. C., et al. (2019). "Determinants and consequences of financial hardship among adult survivors of childhood cancer: A report from the St. Jude Lifetime Cohort Study." Journal of the National Cancer Institute 111(2): 189-200.
20. Huntington, S. F., et al. (2015). "Financial toxicity in insured patients with multiple myeloma: a cross-sectional pilot study." Lancet Haematol 2(10): e408-416.
21. Islam, M. Z., et al. (2021). "Impact of childhood cancer on the family: evidence from Bangladesh." Heliyon 7(2): e06256.
22. Jiang, Q., et al. (2017). "Higher out-of-pocket expenses for tyrosine kinase-inhibitor therapy is associated with worse health-related quality-of-life in persons with chronic myeloid leukemia." Journal of Cancer Research and Clinical Oncology 143(12): 2619-2630.
23. Jones, S. M. W., et al. (2020). "Age and gender differences in financial distress among hematopoietic cell transplant survivors." Supportive Care in Cancer 28(9): 4361-4371.
24. Jones, W. C., et al. (2015). "Prevalence and predictors of distress in posttreatment adult leukemia and lymphoma survivors." Journal of Psychosocial Oncology 33(2): 124-141.
25. Khera, N., et al. (2017). "Prevalence and risk-factors for financial burden in patients with chronic graft-versus-host disease: A cross-sectional survey from the chronic GVHD consortium." Blood 130.
26. Kim, S. H., et al. (2014). "Health-related quality of life in Korean lymphoma survivors compared with the general population." Annals of Hematology 93(9): 1531-1540.
27. Klassen, A. F., et al. (2012). "Immigrant to Canada, newcomer to childhood cancer: A qualitative study of challenges faced by immigrant parents." Psycho-Oncology 21(5): 558-562.
28. Knight, T. G., et al. (2018). "Patient reported financial toxicity in acute leukemia." Blood 132.
29. Knight, T. G., et al. (2020). "Financial toxicity intervention decreases mortality in high risk hematologic malignancy patients." Blood 136(SUPPL 1): 14-15.
30. Knight, T. G., et al. (2020). "Financial toxicity intervention improves quality of life in hematologic malignancy patients." Blood 136(SUPPL 1): 21.

31. Kodama, Y., et al. (2012). "Increased financial burden among patients with chronic myelogenous leukaemia receiving imatinib in Japan: a retrospective survey." BMC Cancer 12: 152.
32. Kopp, M., et al. (2005). "Quality of life in adult hematopoietic cell transplant patients at least 5 yr after treatment: A comparison with healthy controls." European Journal of Haematology 74(4): 304-308.
33. Lansky, S. B., et al. (1983). "Childhood cancer. Medical costs." Cancer 52(4): 762-766.
34. Limburg, H., et al. (2008). "Impact of childhood cancer on parental employment and sources of income: A Canadian pilot study." Pediatric Blood and Cancer 51(1): 93-98.
35. Maheshwari, S., et al. (2021). "Parental outlook and problems faced during lockdown in COVID-19 pandemic: Experience from a paediatric haematology-oncology unit in a developing country-A questionnaire-based survey." Journal of Clinical and Diagnostic Research 15(4): SC01-SC04.
36. McGrath, P. (2016). "'The bills that were coming in…': out of pocket costs during relocation for specialist treatment for haematological malignancies." Supportive Care in Cancer 24(7): 2893-2903.
37. McGrath, P. (2016). "Financial distress during relocation for treatment of a hematological malignancy: Findings for social work." Social work in health care 55(4): 265-279.
38. Meehan, K. R., et al. (2006). "The financial requirements and time commitments of caregivers for autologous stem cell transplant recipients." J Support Oncol 4(4): 187-190.
39. Mostert, S., et al. (2008). "Parental experiences of childhood leukemia treatment in indonesia." Journal of Pediatric Hematology/Oncology 30(10): 738-743.
40. Muffly, L. S., et al. (2016). "Financial toxicity in children, adolescent, and young adult cancer patients and their families: A large national registry analysis from the family reach foundation." Journal of Clinical Oncology 34(15).
41. Pearse, W. B., et al. (2020). "Perceptions of financial and psychosocial burden among patientswith hematologic malignancies treated with novel oralantineoplastic agents." Blood 136(SUPPL 1): 20.
42. Poudyal, B. S., et al. (2020). "A survey in Nepalese patients with acute leukaemia: a starting point for defining financial toxicity of cancer care in low-income and middle-income countries." The Lancet Haematology 7(9): e638-e639.
43. Priscilla, D., et al. (2011). "The socio-demographic and clinical factors associated with quality of life among patients with haematological cancer in a large government hospital in Malaysia." Malaysian Journal of Medical Sciences 18(3): 49-56.
44. Pulivadula Venkatasai, J., et al. (2014). "Assessment of invisible financial burden faced by families with cancer children in a developing country." Pediatric Blood and Cancer 61: S238.
45. Ren, Y. and X. Li (2019). "Direct and indirect costs of families with a child with acute lymphoblastic leukaemia in an academic hospital in China: A cross-sectional survey." BMJ Open 9(7): e030511.
46. Rocha-García, A., et al. (2003). "Out-of-pocket expenditures during hospitalization of young leukemia patients with state medical insurance in two Mexican hospitals." Salud Publica de Mexico 45(4): 285-292.

47. Salas Coronado, D. Y., et al. (2017). "Financial burden and patient-reported outcomes after hematopoietic cell transplantation: Impact of pre-treatment awareness of transplant-associated costs." Blood 130.
48. Sidana, S., et al. (2020). "Quality of life (QOL), financial burden, and perception of care in patients enrolled on clinical trials (CTs)." Journal of Clinical Oncology 38(15).
49. Sidi Mohamed El Amine, B., et al. (2017). "Financial toxicity of the management of multiple myeloma." Haematologica 102: 603.
50. Sneha, L. M., et al. (2017). "Financial burden faced by families due to out-of-pocket expenses during the treatment of their cancer children: An Indian perspective." Indian Journal of Medical and Paediatric Oncology 38(1): 4-9.
51. Van Der Poel, M. W. M., et al. (2014). "Quality of life more impaired in younger than in older diffuse large B cell lymphoma survivors compared to a normative population: A study from the population-based PROFILES registry." Annals of Hematology 93(5): 811-819.
52. Warner, E. L., et al. (2015). "Financial burden of pediatric cancer for patients and their families." Journal of Oncology Practice 11(1): 12-18.
53. Warsame, R. M., et al. (2020). "Financial toxicity and impact on health-related quality of life inpatients with plasma cell disorders." Blood 136(SUPPL 1): 20-21.
54. Xu, R. H., et al. (2020). "Quantifying the Effect of Financial Burden on Health-Related Quality of Life among Patients with Non-Hodgkin's Lymphomas." Cancers (Basel) 12(11).
55. Yu, L., et al. (2017). "Analysis on tyrosine kinase inhibitor discontinuation in patients with chronic myeloid leukemia in the real world: experience from single center." Zhonghua xue ye xue za zhi = Zhonghua xueyexue zazhi 38(9): 754-760.

PRISMA 2020 Checklist

Section and Topic	Item #	Checklist item	Location where item is reported
**TITLE**			
Title	1	Identify the report as a systematic review.	1
**ABSTRACT**			
Abstract	2	See the PRISMA 2020 for Abstracts checklist.	3
**INTRODUCTION**			
Rationale	3	Describe the rationale for the review in the context of existing knowledge.	4
Objectives	4	Provide an explicit statement of the objective(s) or question(s) the review addresses.	4
**METHODS**			
Eligibility criteria	5	Specify the inclusion and exclusion criteria for the review and how studies were grouped for the syntheses.	4, 5
Information sources	6	Specify all databases, registers, websites, organisations, reference lists and other sources searched or consulted to identify studies. Specify the date when each source was last searched or consulted.	4
Search strategy	7	Present the full search strategies for all databases, registers and websites, including any filters and limits used.	4
Selection process	8	Specify the methods used to decide whether a study met the inclusion criteria of the review, including how many reviewers screened each record and each report retrieved, whether they worked independently, and if applicable, details of automation tools used in the process.	5
Data collection process	9	Specify the methods used to collect data from reports, including how many reviewers collected data from each report, whether they worked independently, any processes for obtaining or confirming data from study investigators, and if applicable, details of automation tools used in the process.	5
Data items	10a	List and define all outcomes for which data were sought. Specify whether all results that were compatible with each outcome domain in each study were sought (e.g. for all measures, time points, analyses), and if not, the methods used to decide which results to collect.	5
	10b	List and define all other variables for which data were sought (e.g. participant and intervention characteristics, funding sources). Describe any assumptions made about any missing or unclear information.	5
Study risk of bias assessment	11	Specify the methods used to assess risk of bias in the included studies, including details of the tool(s) used, how many reviewers assessed each study and whether they worked independently, and if applicable, details of automation tools used in the process.	N/A
Effect measures	12	Specify for each outcome the effect measure(s) (e.g. risk ratio, mean difference) used in the synthesis or presentation of results.	N/A
Synthesis methods	13a	Describe the processes used to decide which studies were eligible for each synthesis (e.g. tabulating the study intervention characteristics and comparing against the planned groups for each synthesis (item #5)).	N/A
	13b	Describe any methods required to prepare the data for presentation or synthesis, such as handling of missing summary statistics, or data conversions.	
	13c	Describe any methods used to tabulate or visually display results of individual studies and syntheses.	
	13d	Describe any methods used to synthesize results and provide a rationale for the choice(s). If meta-analysis was performed, describe the model(s), method(s) to identify the presence and extent of statistical heterogeneity, and software package(s) used.	
	13e	Describe any methods used to explore possible causes of heterogeneity among study results (e.g. subgroup analysis, meta-regression).	
	13f	Describe any sensitivity analyses conducted to assess robustness of the synthesized results.	
Reporting bias assessment	14	Describe any methods used to assess risk of bias due to missing results in a synthesis (arising from reporting biases).	9
Certainty assessment	15	Describe any methods used to assess certainty (or confidence) in the body of evidence for an outcome.	N/A

PRISMA 2020 Checklist

Section and Topic	Item #	Checklist item	Location where item is reported
**RESULTS**			
Study selection	16a	Describe the results of the search and selection process, from the number of records identified in the search to the number of studies included in the review, ideally using a flow diagram.	Fig. 1
	16b	Cite studies that might appear to meet the inclusion criteria, but which were excluded, and explain why they were excluded.	
Study characteristics	17	Cite each included study and present its characteristics.	Tab 1.
Risk of bias in studies	18	Present assessments of risk of bias for each included study.	N/A
Results of individual studies	19	For all outcomes, present, for each study: (a) summary statistics for each group (where appropriate) and (b) an effect estimate and its precision (e.g. confidence/credible interval), ideally using structured tables or plots.	N/A
Results of syntheses	20a	For each synthesis, briefly summarise the characteristics and risk of bias among contributing studies.	N/A
	20b	Present results of all statistical syntheses conducted. If meta-analysis was done, present for each the summary estimate and its precision (e.g. confidence/credible interval) and measures of statistical heterogeneity. If comparing groups, describe the direction of the effect.	
	20c	Present results of all investigations of possible causes of heterogeneity among study results.	
	20d	Present results of all sensitivity analyses conducted to assess the robustness of the synthesized results.	
Reporting biases	21	Present assessments of risk of bias due to missing results (arising from reporting biases) for each synthesis assessed.	9
Certainty of evidence	22	Present assessments of certainty (or confidence) in the body of evidence for each outcome assessed.	
**DISCUSSION**			
Discussion	23a	Provide a general interpretation of the results in the context of other evidence.	8,9
	23b	Discuss any limitations of the evidence included in the review.	8,9
	23c	Discuss any limitations of the review processes used.	8,9
	23d	Discuss implications of the results for practice, policy, and future research.	8,9
**OTHER INFORMATION**			
Registration and protocol	24a	Provide registration information for the review, including register name and registration number, or state that the review was not registered.	N/A
	24b	Indicate where the review protocol can be accessed, or state that a protocol was not prepared.	N/A
	24c	Describe and explain any amendments to information provided at registration or in the protocol.	N/A
Support	25	Describe sources of financial or non-financial support for the review, and the role of the funders or sponsors in the review.	4
Competing interests	26	Declare any competing interests of review authors.	2
Availability of data, code and other materials	27	Report which of the following are publicly available and where they can be found: template data collection forms; data extracted from included studies; data used for all analyses; analytic code; any other materials used in the review.	9

From: Page MJ, McKenzie JE, Bossuyt PM, Boutron I, Hoffmann TC, Mulrow CD, et al. The PRISMA 2020 statement: an updated guideline for reporting systematic reviews. BMJ 2021;372:n71. doi: 10.1136/bmj.n71

For more information, visit: http://www.prisma-statement.org/
